# Selenium nanoparticles decorated with *Ulva lactuca* polysaccharide potentially attenuate colitis by inhibiting NF-κB mediated hyper inflammation

**DOI:** 10.1186/s12951-017-0252-y

**Published:** 2017-03-07

**Authors:** Chenghui Zhu, Shuimei Zhang, Chengwei Song, Yibo Zhang, Qinjie Ling, Peter R. Hoffmann, Jun Li, Tianfeng Chen, Wenjie Zheng, Zhi Huang

**Affiliations:** 1 School of Life Science and Technology, Jinan University, Guangzhou, 510632 Guangdong Province China; 2 College of Pharmacy, Jinan University, Guangzhou, 510632 Guangdong Province China; 3 Department of Cell and Molecular Biology, John A. Burns School of Medicine, University of Hawaii, Honolulu, HI USA; 4 College of Chemistry and Material Science, Jinan University, Guangzhou, 510632 Guangdong Province China

**Keywords:** Selenium (Se), Selenium nanoparticles (SeNPs), *Ulva lactuca* polysaccharide (ULP), Inflammatory bowel diseases (IBD), Nuclear factor κ-B (NF-κB)

## Abstract

**Background:**

Selenium (Se) is an essential micronutrient trace element and an established nutritional antioxidant. Low Se status exacerbates inflammatory bowel diseases progression, which involves hyper inflammation in the digestive tract. Se nanoparticles (SeNPs) exhibit anti-inflammatory activity accompanied by low toxicity, especially when decorated with natural biological compounds. Herein, we explored the beneficial effects of SeNPs decorated with *Ulva lactuca* polysaccharide (ULP) in mice subjected to the acute colitis model.

**Results:**

We constructed SeNPs coated with ULP (ULP-SeNPs) in average diameter ~130 nm and demonstrated their stability and homogeneity. Supplementation with ULP-SeNPs (0.8 ppm Se) resulted in a significant protective effect on DSS-induced acute colitis in mice including mitigation of body weight loss, and colonic inflammatory damage. ULP-SeNPs ameliorated macrophage infiltration as evidenced by decreased CD68 levels in colon tissue sections. The anti-inflammatory effects of ULP-SeNPs were found to involve modulation of cytokines including IL-6 and TNF-α. Mechanistically, ULP-SeNPs inhibited the activation of macrophages by suppressing the nuclear translocation of NF-κB, which drives the transcription of these pro-inflammatory cytokines.

**Conclusions:**

ULP-SeNPs supplementation may offer therapeutic potential for reducing the symptoms of acute colitis through its anti-inflammatory actions.

**Electronic supplementary material:**

The online version of this article (doi:10.1186/s12951-017-0252-y) contains supplementary material, which is available to authorized users.

## Background

The micronutrient trace element selenium (Se) is an established nutritional antioxidant. Se carries out its biological effects mainly through the 21st amino acid, selenocysteine, which is incorporated into selenoproteins [1]. Se deficiency has been demonstrated in association with increased risk of chronic inflammatory diseases such as cardiovascular disease and inflammatory bowel diseases (IBD) [2]. IBD is characterized by hyper inflammatory conditions of the colon and small intestine including Crohn’s disease (CD) and ulcerative colitis (UC). Decreased levels of Se have been observed in both UC and CD patients [3]. Moreover, low Se status was found to be associated with exacerbated CD severity and colon cancer risk with an involvement of enhanced epithelial injury [4, 5]. Selenoproteins play important roles in the pathophysiological processes of fine-tuning immunity and inflammatory responses [1]. However, beneficial effects of many other types of dietary and supplemental Se such as Se nanoparticles (SeNPs) remain unclear for diseases like IBD.

SeNPs appear to be more effective than that of other forms of Se at increasing selenoproteins expression, scavenging free radicals, and preventing oxidative DNA damage and have additional benefits such as low toxicity and acceptable bioavailability [6, 7]. Investigations in nanomedicine have shown that nanoparticles decorated with natural biological compounds exhibited therapeutic potential with low adverse effects through specific interactions with target cells [8, 9]. Several strategies to direct nanoparticles into the gut mucosa for treatment of IBD have also been documented, mainly for local (rectal) use [10, 11]. A recent study investigated how drug loaded polymeric nanoparticles targeted the site of inflammation and analyzed the influence of different colon-specific delivery strategies [12]. We have found that some capping agents such as ATP and vitamin C on SeNPs can not only control the size and stability of SeNPs but also enhance cellular uptake and prolong circulation of SeNPs [13]. These effects are apparent despite the similar physical and chemical properties of decorated and undecorated SeNPs compounds and equivalent Se bioavailability [14].

Polysaccharides possess various pharmacological activities, including immune regulation, anti-oxidation, antiviral activities, anti-oncological activity, anti-coagulation, and anti-aging effects. Mounting evidence suggests that fabrication of nanomaterials with bioactive polysaccharide may have several advantages [15, 16]. *Ulva lactuca* polysaccharide (ULP) displays several physicochemical and biological features of interest for food, pharmaceutical, agricultural, and chemical applications. Previous studies have shown that ULP had potent effects on cholesterol lowering, immunomodulatory and anti-heptotoxic property in vivo and in vitro [17, 18]. ULP consisting of rhamnose, xylose, glucose, uronic acid, and sulfate was shown to stabilize the functional status of bio-membranes and act as an antioxidant and surfactant [18–20]. Accordingly, we set out to design SeNPs decorated with ULP and hypothesized that these SeNPs would exhibit anti-inflammatory activity accompanied by low toxicity for functionally attenuating IBD.

In the present study, we constructed ULP-SeNPs of an average diameter ~130 nm. We explored the therapeutic effects of ULP-SeNPs on mice subjected to the DSS-induced colitis mouse model. We also investigated the function of ULP-SeNPs in inhibiting NF-κB activation in macrophages, which represents an important mechanism by which ULP-SeNPs reduce the inflammatory pathology that drives colitis.

## Results

### Preparation and Characterization of ULP-SeNPs

**Fig. 1:**
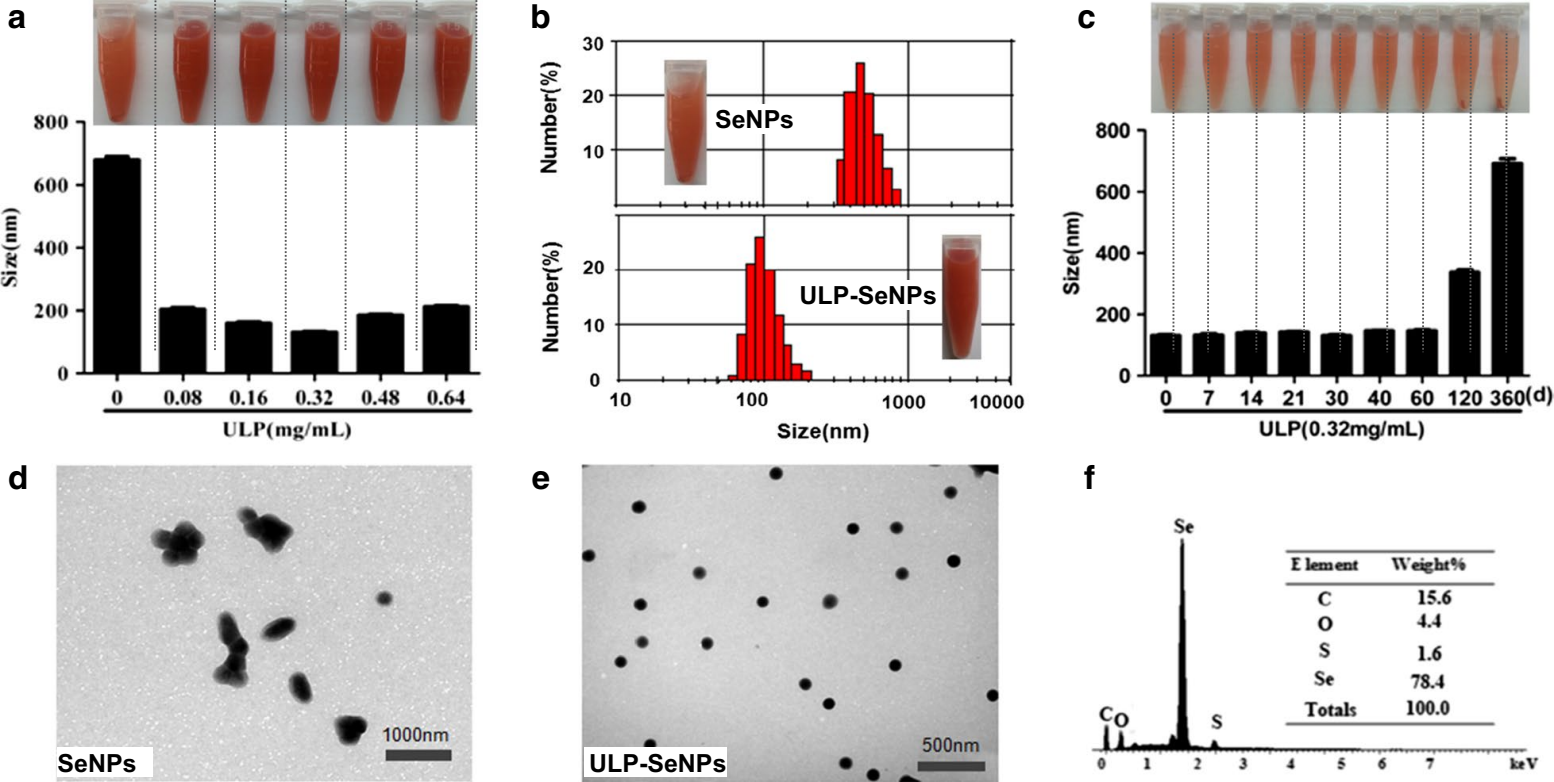
Preparation and characterization of ULP-SeNPs. **a** Particle size of ULP-SeNPs prepared in absence and presence of ULP are shown at indicated concentrations of ULP. **b** Size distribution of SeNPs and ULP-SeNPs. **c** Stability of ULP-SeNPs particle size within the indicated time course, representative TEM images of SeNPs (**d**) and ULP-SeNPs (**e**). **f** Representative EDX analysis of ULP-SeNPs. ULP-SeNPs were obtained at a concentration of 0.32 mg/mL ULP after reacting for 24 h at room temperature in the dark

Nanoparticles with size ranging from 30 to 150 nm were produced to enhance the cellular uptake, with both size and stability being important [21, 22]. Size-controlled SeNPs were prepared in the redox reaction system of selenite acid and ascorbic acid, and for some of these particles we added ULP to generate ULP-SeNPs. The particle size, stability and dispersity of SeNPs and ULP-SeNPs were measured as shown in Fig. 1. SeNPs without ULP decoration showed an average diameter of 680 nm, while addition of 0.32 mg/mL ULP generated ULP-SeNPs with significantly decreased particle diameters to 131 nm (Fig. 1a). The particle-size distribution was 305–900 nm and 58–205 nm for SeNPs and SeNPs-ULP (0.32 mg/mL ULP), respectively (Fig. 1b). There was a trend toward an increase in size of SeNPs with adding ULP above the level of 0.32 mg/mL, which implied that the particle size of SeNPs has something to do with the concentrations of ULP. It was also found that the ULP-SeNPs remained stable at least for 60 days when stored in 0.32 mg/mL ULP solutions (Fig. 1c). Moreover, the TEM images of these nanoparticles clearly revealed that SeNPs tended to aggregate and precipitate in absence of ULP (Fig. 1d), while SeNPs decorated with 0.32 mg/mL ULP appeared to monodisperse into homogeneous spherical structures with an average diameter of 130 nm (Fig. 1e). The diagram from surface elemental composition analysis of ULP-SeNPs by EDX showed four signals, including a strong Se atom signal (78.4%) from SeNPs and minor signals of C (15.6%), O (4.4) and S (1.6%) that likely originated from the ULP (Fig. 1f).

To monitor the physical characteristics of the ULP-SeNPs in solution, we examined the stability of ULP-SeNPs in mouse plasma and in digestion fluid after incubation for 24 h. The average size of ULP-SeNPs was maintained at ~130 nm in both plasma and digestion fluid indicating no aggregation of ULP-SeNPs (Additional file 1: Figure S1A, B). After centrifugation to remove the ULP-SeNPs, dissolved Se levels were only slightly altered in plasma, and Se levels increased by ~16% in the digestion fluid in comparison with controls (Additional file 1: Figure S1C, D). These data imply that the ULP-SeNPs can be deconstructed to some extent in the digestive tract, which is consistent with data below showing increased Se levels in colon and liver of mice supplementated with ULP-SeNPs. To compare the cellular uptake of ULP-SeNPs and SeNPs, intracellular Se concentrations were detected by ICP-MS after BMDMs were incubated with SeNPs or ULP-SeNPs for 24 h. Data showed that incubation of BMDMs with ULP-SeNPs (0.5 μM) significantly increased the intracellular Se concentration from 0.36 to 16.25 ng/10^7^ cells, which was ~2.4 times higher than that of SeNPs treatment (6.9 ng/10^7^cells) (Additional file 1: Figure S2). Taken together, these results indicated that capping ULP on SeNPs not only controls the size and stability of SeNPs but also enhances cellular uptake.

### Effects of ULP-SeNPs on the development of DSS-induced colitis in mice

**Fig. 2:**
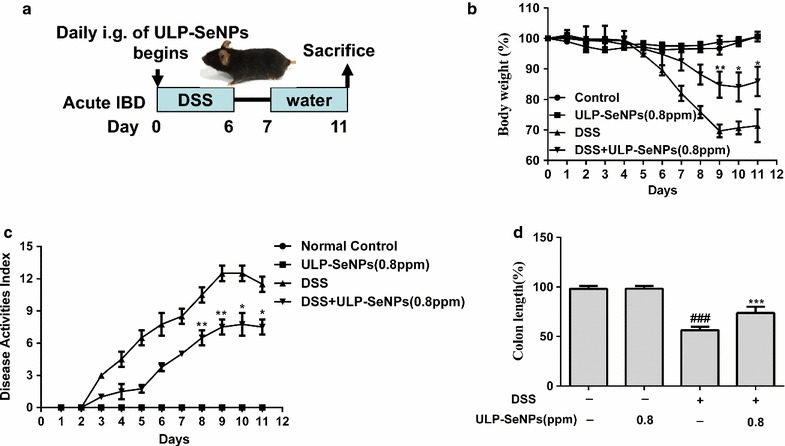
Effects of ULP-SeNPs on DSS-induced mouse colitis. **a** Experimental time lines for acute colitis mouse models induced by DSS with or without ULP-SeNPs, and included negative controls with or without ULP-SeNPs. Groups were compared for body weight changes (**b**), DAI scores (**c**), and colon lengths (**d**), which were measured on day 11 after treatments. Data are mean ± SD (n = 10 mice per group). *P < 0.05; **P < 0.01; ***P < 0.001 (significantly different from the DSS group). ^*###*^ *P* < 0.001 (significantly different from the NC group)

We first explored how ULP-SeNPs influenced inflammation using the mouse model of DSS-induced colitis according to the procedure shown in Fig. 2a. In acute colitis, the mice treated with 0.8 ppm of ULP-SeNPs subjected to colitis (DSS + ULP-SeNPs) exhibited lower body weight (BW) loss, disease activity index (DAI) scores, and colon length (CL) changes compared with those of the DSS group (Fig. 2b–e). In the absence of colitis, administration of ULP-SeNPs to mice induced slight increases in BW and no other general effects on the animal’s health compared to untreated mice. These results suggest that ULP-SeNPs are capable of preventing the clinical manifestations of DSS-induced colitis. Accordingly, the following experiments only included groups of untreated negative controls (NC), DSS, and DSS + ULP-SeNPs.

### Effect of ULP-SeNPs on the colonic damage in DSS-treated mice

**Fig. 3:**
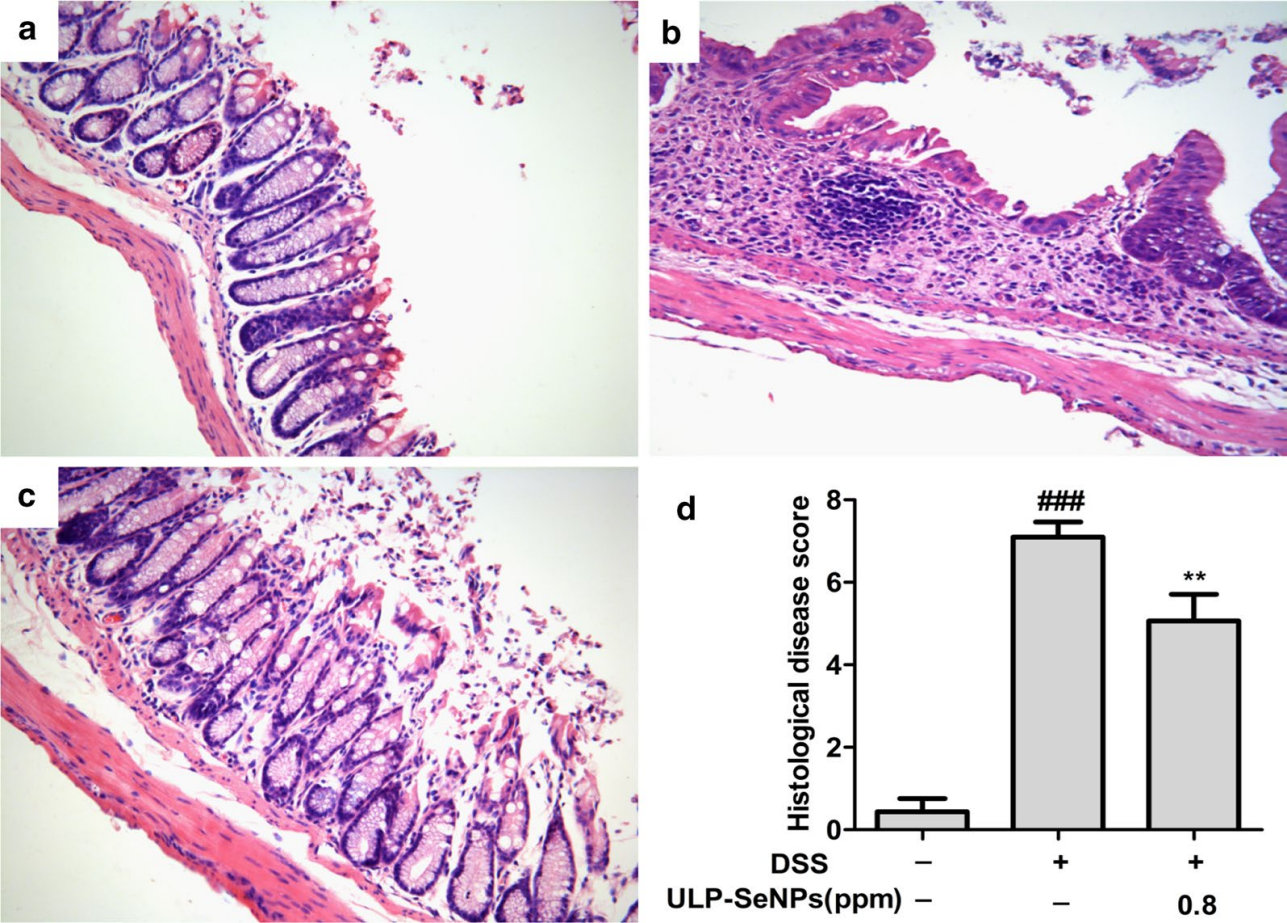
Effects of ULP-SeNPs on histological manifestation in DSS-induced colitis. H&E staining images of representative colons are shown at the same magnification (×200) for NC (**a**), DSS (**b**), and DSS + ULP-SeNPs (**c**). And histological scores were evaluated (**d**). *Each bar chart* represents the mean ± SD (n = 10 animals for per group). **P < 0.01 (significantly different from the DSS group). ^*###*^ *P* < 0.001 (significantly different from the NC group)

To investigate the effects of ULP-SeNPs on colonic pathological changes during colitis, we examined colon tissue sections by H&E staining. The NC group had normal glands, abundant goblet cells in epithelium, no mucosal hyperplasia, and limited levels of infiltrating immune cells (Fig. 3a). In contrast, the DSS-treated group displayed severe histological damage and inflammatory responses, including disruption of epithelial cells, muscle layer thickness, loss of goblet cells and infiltration of inflammatory cells in the colon tissues (Fig. 3b). Compared to the DSS-treated group, the ULP-SeNPs group exhibited decreases in the levels of histological alterations and inflammatory cellular infiltration along with a milder loss of epithelial cells and less expansion of the lesion area in the colon tissues (Fig. 3c). In a manner consistent with the pathological changes, ULP-SeNPs treatment significantly reduced the total histological score of the colitis colon tissues (Fig. 3d).

### Effects of ULP-SeNPs on immune cell infiltration into colon tissue during colitis

**Fig. 4:**
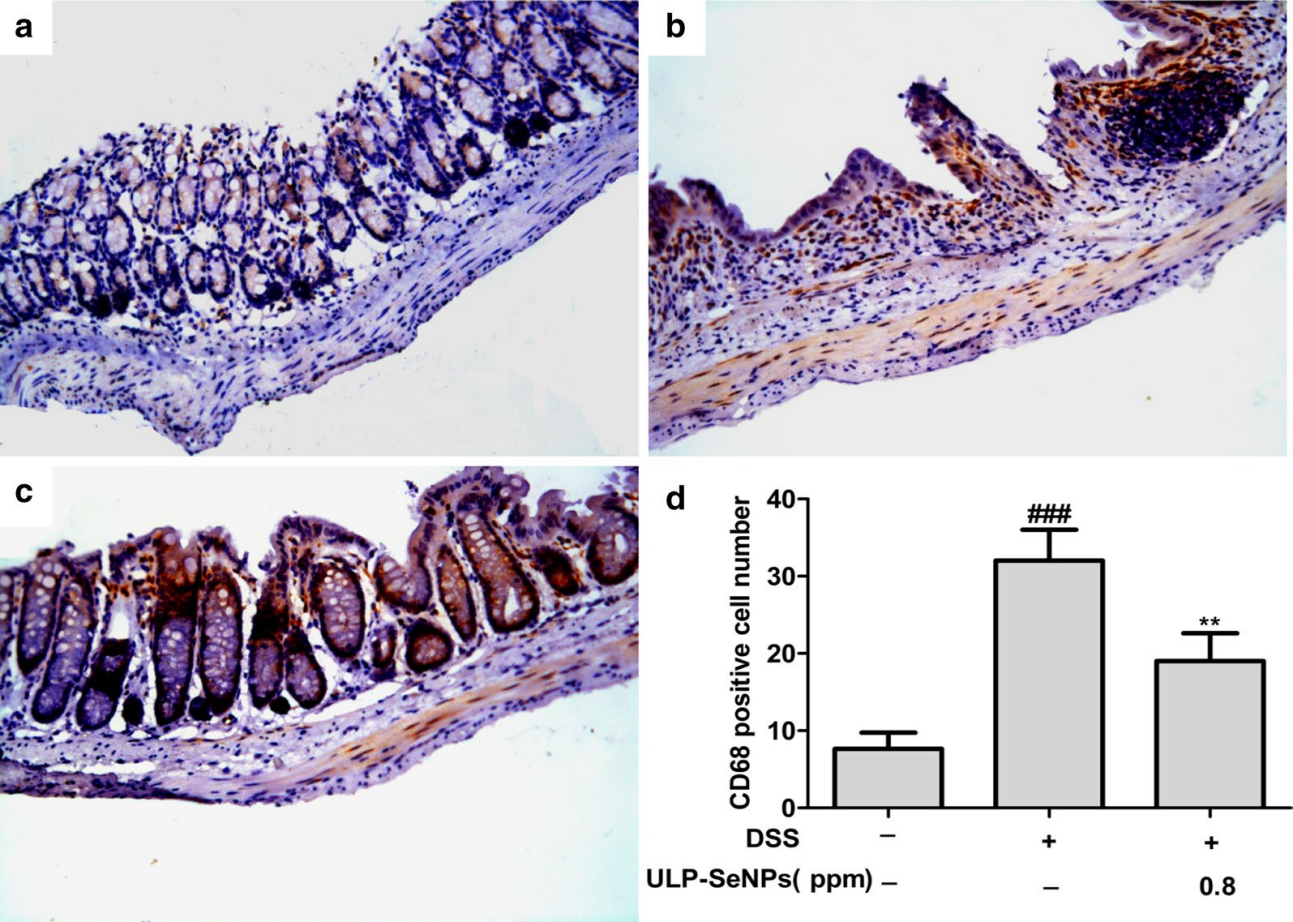
Effects of ULP-SeNPs on immune cell infiltration into colon tissue during DSS colitis. Expression levels of CD68 (×200 magnification) were evaluated by immunohistochemistry in the colon tissue. Three groups including **a** NC, **b** DSS, **c** DSS + ULP-SeNPs and **d** CD68 positive cell numbers were counted and compared among three groups (n = 10). **P < 0.01; (significantly different from the DSS group). ^*###*^ *P* < 0.001 (significantly different from the NC group)

**Fig. 5:**
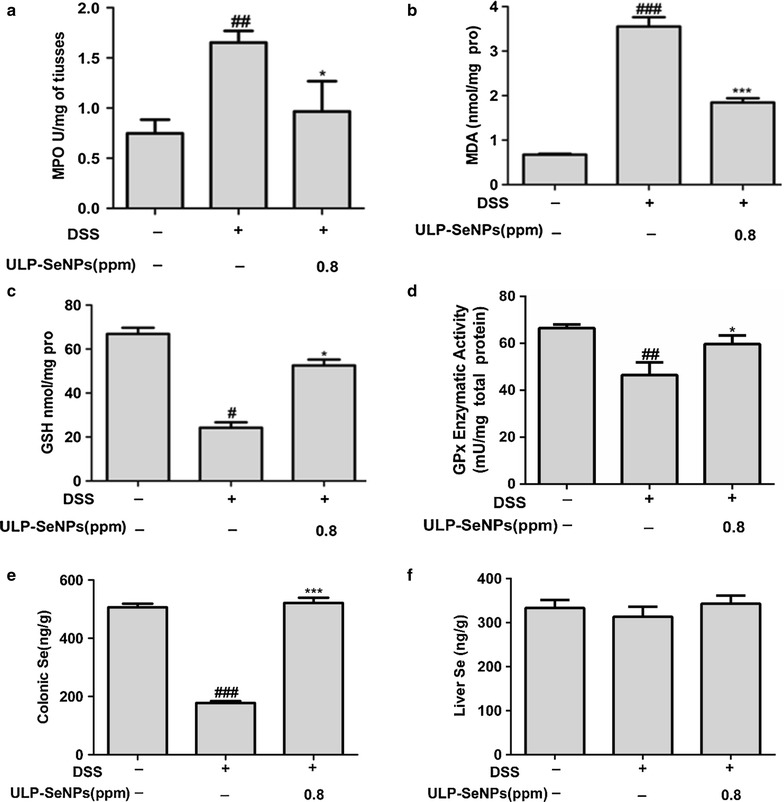
Effects of ULP-SeNPs on the levels of MPO, oxidative stress and Se in DSS-induced colitis. Mice were evaluated for **a** MPO levels, **b** MDA levels, **c** GSH, and **d** GPx activity in colonic tissues. **e** Se levels in colonic tissues and **f** Se levels in livers were evaluated. Data represent mean ± SD (n = 10). *P < 0.05; (significantly different from the DSS group). ^#^ *P* < 0.05, ^##^ *P* < 0.01,^###^ *P* < 0.001 (significantly different from the NC group)

DSS-induced colitis is characterized by substantial immune cell infiltration into colon tissue and myeloperoxidase (MPO) is a biomarker for neutrophil or monocyte/macrophage infiltration [23]. We measured MPO activity in colon tissues of mice with DSS-induced colitis and found the DSS group exhibited high MPO activity (Fig. 5a). The ULP-SeNPs group showed a reduction in MPO activity compared with the DSS group (Fig. 5a). Next, in the colon we determined CD68 expression, which is a specific marker for monocytes/macrophages [24]. The DSS group showed high CD68 expression in lamina propria cells, while the ULP-SeNPs group showed reduced CD68 expression in colon tissues (Fig. 4b, c). These data suggest that ULP-SeNPs inhibit immune cell infiltration in colon tissues during the progression of DSS-induced colitis.

### Effects of ULP-SeNPs on MDA, GSH, GPx and Se levels in DSS-induced colitis

To investigate whether ULP-SeNPs affect oxidative stress, we measured MDA and GSH in the colon tissues of DSS-treated mice with or without ULP-SeNPs. Data showed that DSS significantly increased the colonic MDA levels (3.55 ± 0.21 nmol/mg protein) compared with that of the NC group (0.67 ± 0.20 nmol/mg protein) (Fig. 5b). The ULP-SeNPs group showed significantly reduced colonic MDA levels of 1.84 ± 0.09 nmol/mg protein at 0.8 ppm ULP-SeNPs compared to DSS group (Fig. 5b). In addition, ULP-SeNPs also attenuated the DSS‑induced reduction in colonic GSH levels in the colitis mice (Fig. 5c). We next measured total colonic glutathione peroxidase (GPx) activity to determine the selenoenzyme expression level during colitis DSS lowered GPx activity, but ULP-SeNPs supplementation could reverse this effect (Fig. 5d). Specifically, the colonic Se levels of the ULP-SeNPs group was significantly higher than that of the DSS group (521.09 ± 18.06 vs 177.79 ± 6.38 ng/g), and even slightly higher than that of the NC group (506.06 ± 12.85 ng/g) (Fig. 5e). ULP-SeNPs supplementation also increased liver Se levels in comparison with DSS group (Fig. 5f), implying ULP-SeNPs ameliorated some of the declines in Se status found during DSS induced colitis.

### ULP-SeNPs affect the expression of inflammatory markers in DSS-treated mice

**Fig. 6:**
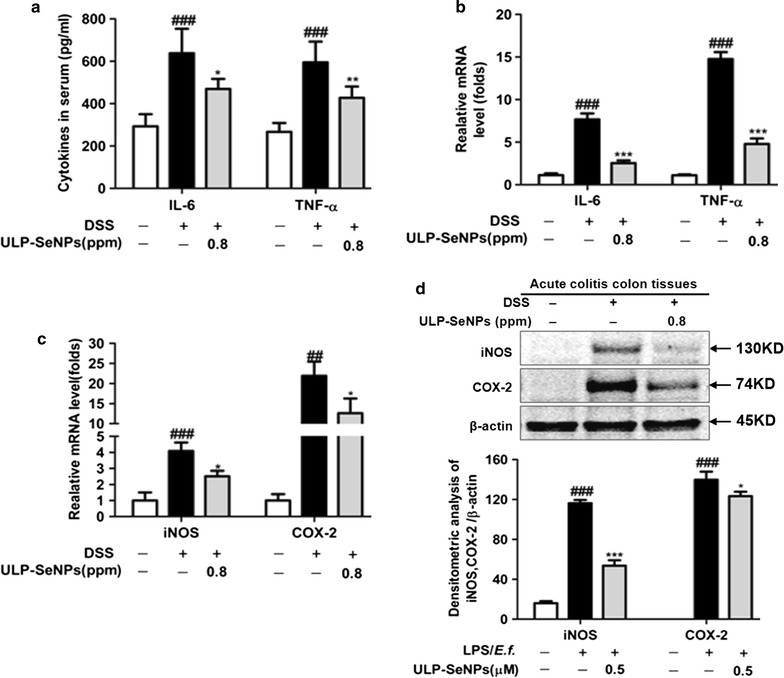
Effects of ULP-SeNPs on inflammatory markers in DSS-induced colitis. **a** Levels of inflammatory cytokines including IL-6 and TNF-α were measured in plasma of DSS-treated mice. **b** Relative mRNA expressions of IL-6 and TNF-α were measured by real-time PCR. **c** Relative mRNA expression of iNOS and COX-2 were measured by real-time PCR. **d** Western blot results for iNOS and COX-2 (*top*) and densitometry (*bottom*). *Each bar chart* represents mean ± SD (n = 10 animals for each experiment). *P < 0.05; **P < 0.01; ***P < 0.001 (significantly different from the DSS group). ^##^ *P* < 0.01; ^###^ *P* < 0.001 (significantly different from the NC group)

Production of pro-inflammatory cytokines is induced by the infiltration of mononuclear cells into the colon during IBD, which contributes to the pathology. The anti-inflammatory effects of ULP-SeNPs were investigated by measuring the inflammatory cytokines in plasma. Compared to NC mice, the levels of inflammatory cytokines in plasma including IL-6 and TNF-α was increased in DSS-treated mice but this effect was mitigated by administration of ULP-SeNPs (Fig. 6a). In addition, we investigated the effects of ULP-SeNPs on mRNA levels for the inflammatory cytokines and on iNOS and COX-2. DSS treatment remarkably increased mRNA levels for TNF-α and IL-6 compared to the NC group, whereas these effects were attenuated by ULP-SeNPs (Fig. 6b, c). Furthermore, the ULP-SeNPs group showed lower COX-2 and iNOS compared to the DSS group (Fig. 6d).

### Inhibitory effect of ULP-SeNPs on the expression of inflammatory mediators in LPS primed and *E.f.* stimulated BMDMs

**Fig. 7:**
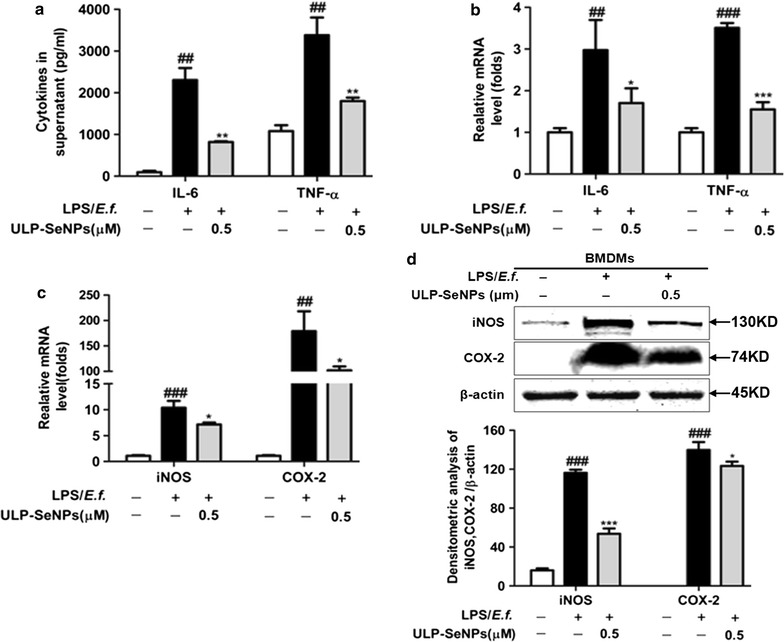
Effect of ULP-SeNPs on of inflammatory marker levels in LPS-primed *E.f.*-activated BMDM. **a** IL-6 and TNF-α levels in cell-free supernatants were determined by ELISA. **b** Relative levels of IL-6 and TNF-α mRNA were measured by real-time PCR. **c** Relative levels of iNOS and COX-2 mRNA were measured by real-time PCR. **d** Analyses iNOS and COX-2 protein by Western blot (*top*) and densitometry (*bottom*). Data represent mean ± SD (n = 3 in independent experiments). *P < 0.05; **P < 0.01; ***P < 0.001 (significantly different from the LPS group). ^##^ *P* < 0.01; ^###^ *P* < 0.001 (significantly different from the control group)

Concentrations of IL-6 and TNF-α were measured in media from macrophages primed with LPS and activated with *E.f.* commensal bacteria in the presence of ULP-SeNPs. Results showed that ULP-SeNPs (0.5 μM) treatment during priming and activation led to a significant decrease in the production of both IL-6 and TNF-α (Fig. 7a). Furthermore, ULP-SeNPs (0.5 μM) had a strong effect on reducing LPS-induced mRNA levels of IL-6, TNF-α, iNOS and COX-2 (Fig. 7b, c). We also found an increased expression of COX-2 and iNOS in LPS primed and *E.f.* activated-macrophages that was markedly reduced by ULP-SeNPs treatment. The densitometry analyses of the immunoblots is shown in Fig. 7d.

### Inhibition of NF-κB activation by ULP-SeNPs

**Fig. 8:**
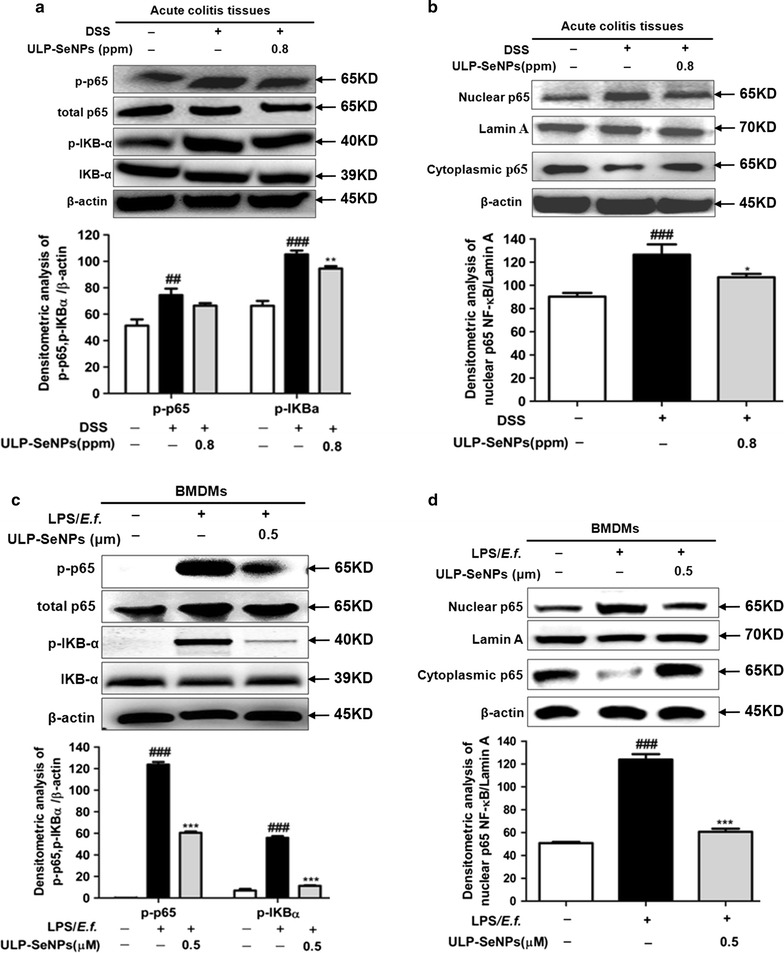
Effect of ULP-SeNPs on NF-κB activation in vivo and in vitro. **a** Effects of ULP-SeNPs on the activation of NF-κB in colon tissues of mice with acute colitis and in BMDMs, as determined by immunoblotting (*top*) and densitometry (*bottom*) analysis. **b** NF-κB levels and nuclear translocation in colon tissues of mice with acute colitis. **c** Effects of ULP-SeNPs on the activation of NF-κB in BMDMs as analyzed by Western blot (*top*) and densitometry (*bottom*) analysis. **d** NF-κB levels and nuclear translocation in BMDM. *P < 0.05; **P < 0.01 (significantly different from the DSS group). ^##^ *P* < 0.01; ^###^ *P* < 0.001 (significantly different from the NC group). ***P < 0.001 (significantly different from the LPS group). ^###^ *P* < 0.001 (significantly different from the control group)

NF-κB regulates the expression of a large number of cytokines and its activation plays a role in IBD [25]. To determine whether ULP-SeNPs affected NF-κB activation, we measured p-p65/p-IKBα in DSS-induced colitis with or without ULP-SeNPs as well as in macrophages activated with inflammatory stimuli. Results revealed that ULP-SeNPs inhibited levels of p-p65/p-IKBα in DSS-induced colitis and macrophages activated with inflammatory stimuli (Fig. 8a, c). Nuclear translocation of NF-κB analyses in mouse colon tissues from the NC, DSS-treated, and DSS + ULP-SeNPs (0.8 ppm) groups revealed that ULP-SeNPs treatment caused a significant reduction in the level of NF-κB activation in acute colitis (Fig. 8b) and in LPS primed macrophages activated with *E. faecalis* (Fig. 8d). These data suggest that ULP-SeNPs are capable of regulating the inflammatory response by inhibiting NF-κB activation.

## Discussion

Multiple factors contribute to the pathogenesis of IBD. The key role of nutrients in IBD has been observed with particular focus on the deleterious effects of the modern “Western diet” that increases the risk of IBD [26]. Micronutrients represent an ideal choice for nutritional intervention against IBD. Se, an essential micronutrient, has attracted a great deal of interest due to its important health effects, particularly the effects related to immune responses and cancer prevention activity [27]. Recent reviews of the role of Se in IBD have concluded that Se status and adequate dietary Se supply are of pivotal importance to colonic inflammation [26, 28]. Nanoparticles that include elemental Se (SeNPs) as a novel form of this nutrient appear to be more effective than that of other Se sources because of their excellent bioavailability, biological activity, and low toxicity [29]. To address application of ULP-SeNPs to prevent IBD, we developed a stable, homogeneous formulation of ULP-SeNPs with consistent diameter of 130 nm. The mouse macrophage reporter cell line-RAW-Blue cells was used to determine the effects of SeNPs and ULP-SeNPs on the LPS/*E.f.* triggered NF-κB inflammatory signaling pathway. Data showed that ULP-SeNPs (0.5 μM) inhibited either LPS or *E.f.*-induced NF-κB hyper-activation, and did so in a more effective manner than ULP or SeNPs (Additional file 1: Figure S3). These data support the notion that ULP-SeNPs may represent a nutriceutical with promising anti-inflammation capacity.

In this study, we report for the first time that ULP-SeNPs led to a reduction in the severity of the systemic (e.g., BW loss and DAI scores) and local (e.g., CL shortened and HDS) symptoms of DSS-induced colitis. Moreover, ULP-SeNPs suppressed inflammatory cell infiltration and local inflammation, resulting in less pathological damage being induced by DSS in colon. DSS-induced acute colitis and chronic colitis in mice reproduce the histological pathology of human IBD [30]. Optimization of dietary ULP-SeNPs supplementation might offer a promising supportive therapy for IBD patients as long as safety can be demonstrated. It is conceivable that ULP-SeNPs would have limited side effects because Se nanoparticles (SeNPs) exhibit excellent bioavailability, biological activity, and low toxicity compared to organic or inorganic Se. Altogether, these results indicate that ULP-SeNPs might be a candidate as a potential therapeutic nanomedicine for prevention of IBD and other inflammatory diseases. However, further follow-up investigations of the safety and efficacy of ULP-SeNPs are necessary before it can be considered for the treatment of IBD patients.

Oxidative stress or cellular damage from free radicals, often involving profound lipid peroxidation are the hallmarks of UC [31]. Mounting evidence suggests that many Se metabolites exhibit antioxidative properties and anti-inflammatory roles for modulating disorders such as IBD, atherosclerosis, and cancer [2, 32]. In the present study, the results showed that ULP-SeNPs markedly increased the colonic GSH levels, one marker for oxidative status, and reduced the generation of MDA to attenuate the DSS-induced colitis in mice (Fig. 5a, b). It should be noted that the diets contained sufficient levels of Se (0.8 ppm) and the ULP-SeNPs were able to further boost antioxidant capacity, indicating potential benefits of ULP-SeNPs in boosting antioxidant capacity. The effects of ULP-SeNPs on systemic and local Se status and particularly on development of IBD are likely complex and nuanced, and our data are suggestive but not conclusive regarding the role of the selenoproteome in the protective actions of Se.

It is well known that the increased pro‑inflammatory cytokines (TNF-α, IL-1β and IL-6) observed in our model of colitis amplify the inflammatory cascade and result in intestinal tissue damage in patients with UC [33]. The downregulation and/or blockade of pro‑inflammatory cytokine activity has been effective in the treatment of IBD [34]. Se at supranutritional levels (as inorganic selenite) affects gene expression, signaling pathways, and cellular functions involved in inflammation However, the underlying mechanisms are not well understood since inorganic Se species and selenoproteins themselves play many roles within cells. In the present study, we focused on ULP-SeNPs that modulate gastrointestinal inflammation, which include macrophages as key regulators. We observed that the colonic levels of TNF-α and IL-6 in the DSS-induced colitis mice were markedly decreased by ULP-SeNPs, as measured by ELISA. The RT-PCR assay confirmed that the mRNA levels of these pro-inflammatory cytokines and the local inflammatory mediator MPO were reduced by ULP-SeNPs. With BMDMs stimulated by LPS primed *E.f.*-stimulated macrophages, we revealed that ULP-SeNPs significantly suppressed mRNA levels of IL-6 and TNF-α. In addition, we demonstrated that ULP-SeNPs decreased the expression of iNOS and COX-2. NF-κB plays a central role in regulating immune and inflammatory processes and thus represents a key target for developing novel treatments for inflammatory diseases [35]. Prior to activation, NF-κB is complexed with IκBα, an inhibitory protein keeping NF-κB inactive state in the cytoplasm. Induced by various stimuli, such as LPS and proinflammatory factors, NF-κB is released and translocates from cytoplasm into the nucleus due to IκBα phosphorylation, ubiquitinylation, and degradation. Phosphorylated subunit p65 plays an important role in triggering the transcription of certain genes. Results in our study showed that ULP-SeNPs strongly inhibited the level of NF-κB activation in colitis colon tissues and stimulated macrophages by suppressing the phosphorylation and degradation of IκBα and thus decreased the phosphorylation of p65. However, inflammation also plays a part in restoring gut homeostasis through diverse pathways involving the colonic macrophage family (e.g. M1 and M2 types), [36] and mediators such as IL-10 and NO. Further mechanistic studies will improve our understanding of the actions of ULP-SeNPs in IBD and identify specific mediators driving the beneficial Se effects demonstrated in our studies.

The data from our in vivo and in vitro experiments suggest that ULP-SeNPs might represent a modality that inhibits IBD. However, our study is limited by a lack of definitive molecules on which the increased Se acts. Altered redox tone in the colon and perhaps modulation of immune cells are two ways that Se has been shown to affect inflammation [1]. The selenoprotein subfamilies of glutathione peroxidases and thioredoxin reductases may be involved in regulating these processes, [37] but other selenoproteins are likely also involved in mediating the effects of SeNPs. Another limitation of our data is that the DSS mouse model of chemically induced colitis is strongly macrophage driven inflammation and not completely representative of human IBD [38]. Several genetic models of colitis have been generated [39] and it would be of interest to repeat these SeNPs experiments in those mice.

## Conclusions

The present data demonstrated that ULP-SeNPs supplementation exhibits the anti-inflammatory effects to reduce the symptoms of acute colitis through inhibition the hyper activation of NF-κB in colonic tissues and macrophages. Therefore, ULP-SeNPs may be a candidate for further evaluation as a potential therapeutic product for treatment IBD and other inflammatory diseases.

## Methods

### Materials and chemicals

DSS was obtained from MP Biomedicals (Illkirch, France). The glutathione (GSH), glutathione peroxidase (GPx) and malondialdehyde (MDA) detection kits were purchased from Nanjing Jiancheng Bioengineering Institute (Nanjing, China). The bicinchoninic acid (BCA) kits for protein assays were supplied by the Beyotime Institute of Biotechnology (Shanghai, China). *Ulva lactuca* polysaccharide was purchased from Elicityl (Grenoble, France). NF-κB activation assay kits were supplied by FIVE photon Biochemicals (San Diego, CA, USA). Dulbecco’s modified Eagle’s medium (DMEM) and the antibiotic mixture (penicillin/streptomycin) were purchased from Invitrogen (Carlsbad, CA, USA). Proteinase inhibitor cocktail and Laemmli buffer were purchased from Bio-Rad (Hercules, CA, USA). Antibodies purchased from Cell Signaling Technology (Danvers, MA, USA) included anti-NF-κB(p65), anti-p-p65, anti-p-IKBα, anti-IKBα, anti-iNOS, anti-COX-2, anti-β-actin, anti-lamin A. Secondary antibodies for western blots were obtained from Li-Cor (Lincoln, Nebraska, USA). Anti-CD68 was purchased from Santa Cruz Biotechnology (Dallas, Texas, USA). Hexadecyltrimethylammonium bromide, o-dianisidine, and lipopolysaccharide (LPS) purified from Escherichia coli O111:B4 were purchased from Sigma-Aldrich (St. Louis, MO, USA). Heat-killed *Enterococcus faecalis* (*E.f.*) was obtained from American Type Culture Collection. All of the solvents used were of high performance liquid chromatography (HPLC) grade. Chemicals used were of analytical grade, including sodium selenite, sodium dodecyl sulfate (SDS), Tween 20, and other commonly used reagents were obtained from Guangdong Guanghua Sci-Tech (Huada & JHD, Guangzhou, China). The ultrapure water used in all experiments was supplied by a Milli-Q water purification system from Millipore (Bedford, MA, USA).

### Preparation of ULP-SeNPs

The solution of ULP was prepared by dissolving 1 g of ULP powder in 100 mL of Milli-Q water. The solution of 40 mM ascorbic acid was freshly prepared. As a typical procedure, varied volume of ULP solution was added dropwise into sodium selenite solution (1 mL, 100 mM) under magnetic stirring, and then 10 mL of 40 mM ascorbic acid solution was added into the mixture, and it was reconstituted to a final volume of 20 mL with Milli-Q water. The final concentration of Se was 5 mM, and the reactant concentrations of ULP were 0, 0.08, 0.16, 0.32, 0.48 and 0.64 mg/mL. The solution was dialyzed against Milli-Q water until no Se was detected in the outer solution by ICP-MS analysis.

### Characterization of ULP-SeNPs

The ULP-SeNPs were characterized by microscopic and spectroscopic methods including transmission electron microscopy (TEM), energy dispersive X-ray (EDX), dynamic light scattering (DLS). Briefly, TEM was carried out with a Hitachi (H-7650) at an acceleration voltage of 80 kV. The EDX were taken on a JEOL 2010 high-resolution TEM operated at 200 kV. A Zetasizer Nano ZS particle analyzer (Malvern Instruments Limited) was used to measure the particle size, size distribution, and stability of the nanoparticles in aqueous solution, plasma and digestion fluid by DLS measurement.

### Animals and induction of DSS-induced IBD

Male C57BL/6 J mice (6–8 weeks old), SPF bedding, and certified diet were purchased from the Sun Yat-Sen University Animal Breeding Unit (Guangzhou, China). All animals were fed standard laboratory chow housed in wire cages at 21 ± 3 °C and 55 ± 15% relative humidity with a 12 h light/dark cycle, and allowed water ad libitum. The Se content of the diet was 0.12 ± 0.01 ppm on average.

Acute colitis was induced by oral administration of 4% (w/v) DSS dissolved into drinking water, for 7 days according to the method employed in previous studies [40]. A cohort of mice was randomly divided into three groups (n = 10 per group) for acute treated as a normal control group (NC), a DSS-induced colitis group (DSS), and a DSS with 0.8 ppm of ULP-SeNPs treatment group (DSS + ULP-SeNPs). Body weights (BW) were measured daily. The daily weight changes were calculated by percent in relation to the initial weight measured. After 11 days, mice in each acute cohort had blood and colon tissues analyzed after being humanely euthanized. Blood was collected in a heparinized tube, immediately centrifuged to separate plasma, and then kept at −80 °C. The colon was rapidly excised and placed in 4% paraformaldehyde for histological assessment. The remaining colon samples were flash frozen and stored at −80 °C until further analysis.

### Assessment of severity of colitis

A general assessment of colitis was performed as described previously [41]. Briefly, the severity of the colitis was measured by evaluating the disease activity index through the scoring of weight loss, stool consistency, bleeding and coat roughness (grade from 0 to 4 on severity of each index), general activity and bedding contamination by stool and blood (graded from 0 to 2 on severity of each index).

### Histological analysis

Colon tissues were collected, fixed in 4% paraformaldehyde, subjected to consecutive steps of alcohol–xylene changes, and embedded in paraffin from which 5 μm Sections were prepared. Standard hematoxylin and eosin (H&E) staining of paraffin-embedded tissue samples was conducted as previously described [42]. Five H&E-stained sections from each mouse were scored blindly, as previously described [43]. Images were captured using a Zeiss Axioskop 2 plus upright light microscope and camera (Zeiss, Oberkochen, Germany).

### IHC staining

Parafin-embedded tissues were cut into 5 μm-thick sections, deparaffinized in xylene, and rehydrated in graded ethanol. Sections were incubated with anti-CD68 antibodies overnight at 4 °C and then incubated with SignalStain^®^Boost IHC Detection Reagent (HRP, Rabbit) as indicated by the manufacturer (Cell Signaling Technology, Danvers, MA, USA) for 30 min at room temperature. Finally, the sections were stained with DAB + Liquid according to manufacturer’s protocol (DAKO, Glostrup, Denmark).

### Determination of Se

Se concentration in solutions and biological samples was determined by an inductively coupled plasma mass spectrometry (ICP-MS) method described previously [44]. To prepare liver and colonic tissues for this method, the tissues were weighed and mineralized in HNO_3_ 65% (ICP-MS grade) and then diluted 100 fold in deionized water.

### MPO assay

MPO activity, a marker of polymorphonuclear neutrophil primary granules, was measured in colonic tissues using the method by Bradley et al. [45]. Briefly, colon segments were homogenized at 50 mg/mL in phosphate buffer (50 mmol/L, pH 6.0) with 0.5% hexadecyltrimethylammonium bromide. Samples were centrifuged at 30,000×*g* for 15 min at 4 °C after freezing and thawing 3 times. The supernatants were diluted 1/30 with 50 mmol/L phosphate buffer (pH 6.0) containing 0.167 mg/mL o-dianisine (Sigma) and 0.0005% H_2_O_2_. Changes in absorbance between 1 and 3 min at 450 nm were measured with a spectrophotometer. MPO was expressed in units per milligram of wet tissue.

### Glutathione content, GPx activity and lipid peroxidation assay

Colon tissue (100 mg) was washed and homogenized in cooled PBS. The samples were then homogenized followed by centrifugation at 3000 rpm for 15 min. Supernatant was collected for measuring total GSH, GPx activity, and lipid peroxidation of MDA using GSH, GPx and MDA detection kits, respectively. All assays were conducted according to the manufacturer’s instructions. The protein content was assayed by bicinchoninic acid (BCA) kits.

### Cell culture

Bone marrow-derived macrophages (BMDMs) were prepared as previously described [46]. On day 5 of culture, BMDMs were plated at a density of 2 × 10^6^ cells/well in six well plates in 2 mL DMEM with 5% FBS (Life Technologies, Gaithersburg, MD) penicillin (100 U/mL), and streptomycin (50 U/mL) overnight at 37 °C in a humidified incubator with a 5% CO_2_ atmosphere. The next day, cultured cells were pretreated with ULP-SeNPs at the indicated concentrations for 1 h and then were primed for 18 h with LPS (100 ng/mL), followed by 1 h stimulation with *E. f.* (1 μg/mL). Supernatants of the cultures were collected at the indicated time point for cytokine detection.

### RNA isolation, cDNA synthesis, and real-time PCR

To detect the effect of ULP-SeNPs on gene expression in colon tissues and LPS primed and *E.f.*-stimulated cells, Tissue samples were thawed and RNA extracted using RNeasy Mini kit and RNase-free DNase I (Qiagen, Hilden, Germany), in accordance with the manufacturer’s instructions. BMDMs were preincubated on six-well plates (1 × 10^6^ cell/mL) and were pretreated with ULP-SeNPs (0.5 μM) 1 h prior to LPS (100 ng/mL) primed and *E. f*. (1 μg/mL) treatment in a 37 °C, 5% CO_2_ incubator for 12 h. Total RNA from BMDMs was performed using RNeasy (Qiagen, Hilden, Germany) according to the manufacturer’s instructions. The quantity and quality of RNA were determined by Nano Drop ND-1000 (Thermo Scientific, Waltham, MA, USA). The cDNA was synthesized using Superscript III (Invitrogen, Carlsbad, CA, USA) and oligo dT primer, with 2 µg of total RNA per 50 µL reaction. For real-time PCR, 1 µL of this cDNA was used in 10 µL reactions with Platinum SYBR Green qPCR SuperMix-UDG (Invitrogen). Reactions were carried out in a 9700HT thermal cycler (Applied Biosystems, Foster City, CA) using the following conditions: denaturation for 2 min at 50 °C, for 2 min at 95 °C, and 40 cycles of 95 °C at 15 s, 60 °C at 60 s. The mouse primers used were as follows: IL-6 (forward: 5′-AGAAGGAGTGGCTAAGGACCAA-3′; reverse: 5′-ACGCACTAGGTTTGCCGAGTA-3′), β-actin (forward: 5′-GCATTGTTACCAACTGGGACGA-3′; reverse: 5′-TGGCTGGGGTGTTGAAGGTC-3′), TNF-α forward: (5′-AGGACCCAGTGGTGGGAAGCT-3′; reverse: 5′-AAAGAGGAGGCAAGGTAGAGA-3′), IL-10 (forward: 5′-GCTCTTACTGACTGGCATGAG-3′; reverse: 5′-CGCAGCTCTAGGAGCATGTG-3′), COX-2 (forward: 5′-TTGCTGTACAAGCAGTGGCAAAGG-3′; reverse: 5′-AGGACAAACACCGGAGGGAATCTT-3′), iNOS (forward: 5′-CAGATCGAGCCCTGGAAGAC-3′; reverse: 5′-CTGGTCCATGCAGACAACCT-3′). Relative gene expression was calculated according to the $2^{{ - \Delta \Delta {\text{C}}_{\text{t}} }}$ method [47] using mouse β-actin as an endogenous control for normalization. Results were expressed as fold changes relative to the negative control samples.

### Enzyme-linked immunosorbent assay

Plasma samples and cell supernatants were analyzed for levels of inflammatory cytokines including TNF-a, IL-6 and IL-10 using commercially available enzyme-linked immunosorbent assay (ELISA) kits (R&D Systems Inc, USA), according to the manufacturer’s instructions.

### Western blot analysis

Cells pellets were harvested and lysed in lysis buffer [150 mM NaCl, 5 mM EDTA, 50 mM Tris–HCl (pH 7.4), 1% Triton X-100, and 0.5% sodium deoxycholate] with a 1× proteinase inhibitor cocktail. Colon tissues were homogenized with lysis buffer on ice and then centrifuged at 13,000×*g* for 15 min at 4 °C. In some cases, nuclear and cytosolic fractions were separated from cells or colon tissues using the NF-κB activation assay kits. After separation on a 12% polyacrylamide gel, proteins were transferred to polyvinylidene difluoride (PVDF) membranes (Millipore) and then incubated using specific antibodies to detect target proteins. After the primary antibody reaction overnight at 4 °C, the PVDF membrane was washed three times with TBST (137 mM NaCl, 2.7 mM KCl, 19 mM Tris base, and 0.1% Tween 20) and incubated with secondary Abs from Li-Cor for 1 h. Membranes were washed with TBST, and signals were detected with densitometry conducted using the Odyssey imaging system (Li-Cor, Lincoln, NE). Densitometry of blots was analyzed and normalized by β-actin or lamin A using ImageJ (National Institutes of Health, Bethesda, MD).

### Statistical analysis

All comparison analyses were conducted using the SPSS statistical software package (SPSS 13.0 for Windows, SPSS, Inc., Chicago, IL). A P value of <0.05 was considered statistically significant. Comparison of mean values was done using analysis of variance (ANOVA). For the body weight change, DAI scores and colon lengths were analyzed using one-way ANOVA (Welch ANOVA in cases of unequal variance) followed by the Games–Howell post hoc test. All other analyses of equal sample sizes for each group and equal variances among the groups were compared using one-way ANOVA with the Tukey post hoc test.

## Additional file

**Additional file 1.** Supplemental information of ULP-SeNPs concerns their stability in physiological solutions, uptake by BMDMs and effect on NF-κB activation.

## Abbreviations

SeNPs: selenium nanoparticles
Se: selenium
ULP: *Ulva lactuca* polysaccharide
IBD: inflammatory bowel disease
DSS: dextran sulfate sodium
IL-6: interleukin-6
IL-10: interleukin-10
TNF-α: tumor necrosis factor-α
NF-κB: nuclear factor κ-B
p-NF-κB: phospho-nuclear factor κ-B
p-IKB-α: phospho-IKB-α
UC: ulcerative colitis
CD: Crohn’s disease
BCA: bicinchoninic acid
SDS–PAGE: sodium dodecyl sulfate–polyacrylamide gel electrophoresis
DAI: disease activity index
H&E: hematoxylin and eosin
MPO: myeloperoxidase
GSH: glutathione
MDA: malondialdehyde
ELISA: enzymelinked immunosorbent assay
GSH: glutathione
FBS: fetal bovine serum
LPS: lipopolysaccharide
*E.f.*: *Enterococcus faecalis*
iNOS: inducible nitric oxide synthase
SD: standard deviation
