# Supplementary material for: Selenium nanoparticles decorated with Ulva lactuca polysaccharide potentially attenuate colitis by inhibiting NF-κB mediated hyper inflammation
Source: J Nanobiotechnology. 2017 Mar 7;15:20. doi: 10.1186/s12951-017-0252-y (PMC5341357; doi:10.1186/s12951-017-0252-y)
Supplement: Supplementary file 1 — Additional file 1. Supplemental information of ULP-SeNPs concerns their stability in physiological solutions, uptake by BMDMs and effect on NF-κB activation. [file 12951_2017_252_MOESM1_ESM.docx]

**Supplemental Data**

Selenium Nanoparticles Decorated with *Ulva lactuca* Polysaccharide Potentially Attenuate Colitis by Inhibiting NF-κB Mediated Hyper Inflammation

Chenghui Zhu, Shuimei Zhang, Chengwei Song, Yibo Zhang, Qinjie Ling, Peter R. Hoffmann, Jun Li, Tianfeng Chen, Wenjie Zheng, Zhi Huang*

**Results**

**1. Stability and Se Dissolve of ULP-SeNPs in Physiological Solutions**

To monitor the physical characteristics of the ULP-SeNPs in physiological solutions, we examined the stability and Se dissolve of ULP-SeNPs in mouse plasma and digestion fluid after incubation for 24 h. The average size of ULP-SeNPs was maintained around 130 nm in both plasma and digestion fluid (Figure S1 A, B), indicating no aggregation of ULP-SeNPs. After centrifugation to remove the ULP-SeNPs, dissolved Se levels was changed slightly (less than 5%) in plasma, and which was increased ( ~ 16 %) in the digestion fluid in comparison with controls (Figure S1 C, D).

**2. Se uptake by BMDMs with treatments of ULP-SeNPs or SeNPs**

To compare the cell uptake of ULP-SeNPs and SeNPs, intracellular Se concentration was detected by ICP-MS after BMDMs incubated with SeNPs or ULP-SeNPs for 24 h. Data showed that treatments of BMDMs with ULP-SeNPs (0.5 μM) significantly increased the intracellular Se concentration from 0.36 up to 16.25 ng/10^7^ cells, which was about 2.4 times higher than that of without ULP decoration SeNPs treatment (6.9 ng/10^7^ cells) (Figure S2).

**3. Inhibitory Effect of ULP-SeNPs on NF-κB hyper-activation**

Using the mouse macrophage reporter cell line-RAW-Blue cells, we performed a screening assay to determine the effects of SeNPs, ULP and ULP-SeNPs on the LPS and *E.f.* triggered TLRs/ NF-κB inflammatory signaling pathway. Data showed that ULP-SeNPs (0.5μM) treatments inhibited the LPS or *E.f.*-induced NF-κB hyper-activation, which was much more efficiency than that of ULP or SeNPs (Figure S3).

**4. Western blots of COX-2, pIkB, tIkB and β-actin**

Western blots with the molecular weight (MW) standards were obtained by the Odyssey Li-Cor Scanner imaging system. The targets of COX-2 (74 kDa), pIkB (40 kDa), tIkB (39 kDa) and β-actin (45 kDa) were shown in green colored bands, whereas the indicated MW standard near with the targets on the 'cut membrane' scanning image were shown in red (Figure S4 A and B).

**Materials and methods**

**1. Stability and Se Dissolve of ULP-SeNPs in Physiological Solutions**

ULP-SeNPs at Se concentration of 0.5 μM were incubated with mouse plasma and digestion fluid for 24 h, respectively. Stability of the ULP-SeNPs in physiological solutions was monitored by dynamic light scattering (DLS). A Zetasizer Nano ZS particle analyzer (Malvern Instruments Limited) was used to measure the average particle size, the size distribution, and the stability of the nanoparticles in plasma and digestion fluid.

After incubation, nanoparticals were removed by centrifugation, pipetted carefully the supernatant , and then detected the dissolved Se levels in plasma and in the digestion fluid by an inductively coupled plasma mass spectrometry (ICP-MS) method described previously [1]. Samples were mineralized in HNO3 65% (ICP-MS grade) and diluted in deionized water prior to ICP-MS determination.

**2. Se uptake by BMDMs with treatments of ULP-SeNPs or SeNPs**

BMDMs (10^7^ cells) were incubated with SeNPs or ULP-SeNPs (at Se concentration of 0.5 μM) for 24 h. After incubation, cells were washed twice quickly with pre-cooled PBS and centrifugated (500 ×g) at 4 ^o^C for 5 min, and then the intracellular Se concentration (represent the uptake of Se by BMDMs) were detected by ICP-MS according to the ICP-MS method described as above.

**3. SEAP reporter assay**

RAW-Blue™ cells (Invitrogen, San Diego, CA) are derived from RAW264.7 macrophages with chromosomal integration of a embryonic alkaline phosphatase (SEAP) reporter construct inducible by NF-κB. According to the manual instruction, the RAW-Blue™ cells were cultured in DMEM supplemented with 10% (v/v) heat-inactivated FBS and zeocineosin (200 μg/mL). The cell suspension (1×10^5^ cells/well in 200 μL) were seeded in a 96-well plate and pretreated with ULP (0.32 mg/ml), SeNPs and ULP-SeNPs (at Se concentration of 0.5 μM) for 1 h at 37 °C. After washing twice in PBS, the cells were treated with 10^7^ bacteria/well heated killed *Enterococcus faecalis* (*E. f.*) or 1 μg/ml LPS for 18 h. The supernatants were collected for SEAP secretion assay. QUANTI-Blue™ powder was dissolved in endotoxin-free water and sterile filtered (0.22 μm) to produce a QuantiQuanta-blue substrate. The cell supernatant (50 μl/well) were added to the substrate (150 μl/well) and incubated at 37 °C for 1 h. Absorbance was measured at 630 nm by an ELISA plate reader (Synergy H1,BioTek, Winooski, VT).

**4. Western blots of COX-2, pIkB, tIkB and β-actin**

Western blots with the molecular weight (MW) standards were obtained by the Odyssey Li-Cor Scanner imaging system. To do the Western blot, we run the gel, transfer separated proteins on PVDF membrane, and then cut the PVDF membrane to several pieces by the predict MW of the targets to detect different goals. Western blots were performed standard method. In briefly, membranes were incubated with specific primary antibody respectively overnight at 4 °C, then washed three times with TBST and incubated with secondary Abs from Li-Cor for 1 h. Membranes were washed again with TBST, and scanned by the Odyssey imaging system (Li-Cor, Lincoln, NE).

**Reference**

Huang Z, Pei QL, Sun GF, Zhang SC, Liang J, Gao Y, Zhang XR. Low selenium status affects arsenic metabolites in an arsenic exposed population with skin lesions. Clinica Chimica Acta, 2008, 387(1-2): 139-144.

**Figure Legend**

**Figure S1**

**Stability and Se Dissolve of ULP-SeNPs in Physiological Solutions.** The average size of ULP-SeNPs in plasma (A) and in the digestion fluid (B); Dissolved Se levels in plasma (C), and in the digestion fluid (D) in comparison with controls. ULP-SeNPs were incubated in mouse plasma and digestion fluid for 24 h.

**Figure S2**

**Se uptake by BMDMs with treatments of ULP-SeNPs or SeNPs.** Intracellular Se concentration of BMDMs (10^7^ cells) treated with ULP-SeNPs (0.5 μM) for 24h was determined and compared with SeNPs treatment.

**Figure S3**

**Inhibitory Effect of ULP-SeNPs on NF-κB hyper-activation.** Effects of SeNPs, ULP and ULP-SeNPs treatments on the LPS and *E.f.* triggered NF-κB hyper- activation and inflammatory response. Screening assay was performed using the mouse macrophage reporter cell line-RAW-Blue cells.

**Figure S4**

**Western blots of COX-2, pIkB, tIkB and β-actin.** Western blots of COX-2 and β-actin (A), pIkB and tIkB (B) were obtained by the Odyssey Li-Cor Scanner imaging system. The targets of COX-2 (74 kDa), pIkB (40 kDa), tIkB (39 kDa) and β-actin (45 kDa) were shown in green, whereas the indicated MW standard were shown in red.

**Figure S1**

**Figure S2**

**Figure S3**

**Figure S4**
